# The Gentlemen’s Club: An innovation to improve HIV counselling and testing uptake at a South African university campus

**DOI:** 10.4102/phcfm.v5i1.455

**Published:** 2013-04-08

**Authors:** Sogo F. Matlala, Selokela J. Mokono, Pontsho Tsotetsi

**Affiliations:** 1 Department of Medical Science, Public Health and Health Promotion, University of Limpopo Turfloop Campus, South Africa; 2 Student Health Centre, University of Limpopo Turfloop Campus, South Africa

## Abstract

**Background:**

Many university students were found to be engaging in high HIV risk practices on campuses which then necessitated discovering their HIV status by participating in HIV counselling and testing (HCT) campaigns. HCT is an entry point into a comprehensive continuum of prevention, treatment, care and support services for HIV infection and AIDS. However, it was also found that many students, mostly males, did not take regular HIV tests to discover their HIV status and receive the necessary counselling and support.

**Objectives:**

The innovative Gentlemen’s Club was therefore implemented at a university campus to increase HCT uptake.The club was formed to motivate male students on behaviour and lifestyle changes so that they could become responsible men.

**Method:**

To join the club, a student was required to take a confidential HIV test and as a member he was expected to follow rules of good and responsible behaviour as prescribed by the club.

**Results:**

Club membership and attendance for meetings showed an increase after the launch of the club in 2010 because of its appeal, and there was also a notable increase in the number of male students attending HCT campaigns. Women have formed a similar club to motivate other women to take regular HIV tests as well.

**Conclusion:**

The Gentlemen’s Club was an innovative idea that increased HCT uptake by male students and served as vehicle to address health and social issues facing university students.

## Abstrait

Les Gentlemen Club: Une innovation pour améliorer le conseil et le taux de test sur ​​un campus université sud-africaine

**Présentation:**

Bon nombre d’étudiants en université prenaient part à des pratiques augmentant le risque de transmission ou de contraction du VIH sur les campus, les obligeant ensuite à découvrir leur statut sérologique en prenant part à des campagnes de conseil et dépistage du VIH (CDV). Le CDV constitue un point d’accès à une gamme complète de services de prévention, de traitement, de prise en charge et de soutien associés au VIH et au Sida. Mais de nombreux étudiants, pour l’essentiel de sexe masculin, ne se font pas régulièrement dépister pour découvrir leur statut sérologique et ne bénéficient donc pas des conseils et du soutien nécessaire.

**Objectifs:**

Le Gentlemen’s Club, un concept novateur, a donc été créé sur un campus universitaire en vue d’augmenter l’adhésion au CDV. Le club a été formé afin de motiver les étudiants de sexe masculin à changer de comportement et de mode de vie et de devenir des hommes responsables.

**Méthode:**

Pour pouvoir adhérer au club, un étudiant devait passer un test de dépistage du VIH anonyme et, en tant que membre, il devait suivre les règles de comportement responsable prescrites par le club.

**Résultats:**

L’adhésion au club et la participation aux réunions ont connu une augmentation après le lancement du club en 2010 en raison de son caractère attractif; on a également pu observer une augmentation notoire du nombre d’étudiants de sexe masculin participant aux campagnes de CDV. Les étudiantes ont formé un club similaire afin de motiver d’autres femmes à se faire également dépister régulièrement.

**Conclusion:**

Le Gentlemen’s Club constitue une idée novatrice qui a permis d’augmenter l’adhésion des étudiants de sexe masculin au CDV et a constitué un moyen de s’attaquer aux problèmes sanitaires et sociaux auxquels les étudiants en université se trouvent confrontés.

## Introduction

There is a high level of HIV risk practices amongst university students worldwide,^1^ necessitating a need for them to know their HIV status.^2^ Taking an HIV test is a way to know one’s HIV status but few male university students take the test. This case study reports on an innovative idea implemented at a university campus to motivate male students to take regular HIV tests and live responsibly.

## Case presentation

HIV counselling and testing (HCT) uptake amongst students at the University of Limpopo Turfloop Campus is low and more females than males utilise the available reproductive health services, as is the case in the general population in South Africa.^3,4^ Nationally and internationally male HIV testing continues to lag behind that of females.^5^ HCT is very important as it is an entry point into a comprehensive continuum of care involving prevention, treatment, care, support and wellness regarding HIV infection and the subsequent development of AIDS.^3,6,7^ HCT, which in South Africa was previously referred to as VCT, enables people to know their HIV status, which in turn leads to healthier lifestyles such as following safe sex practices, and a reduction in HIV-associated denial as well as in stigmatising and discrimination.^8,9^ People who test positive are referred for further tests to evaluate their CD4 count in preparation for life-sustaining ARV treatment and care programmes.^10,11^ Communication and mobilisation strategies for HIV and AIDS prevention, treatment and care services can convince people of the importance of taking an HIV test regularly.^12^ When people become used to the available communication and mobilisation strategies, however, innovation becomes necessary.

## Management and outcome

The Gentlemen’s Club was formed to motivate male students to implement behaviour and lifestyle changes so that they become responsible men. The club is an innovative way to reinforce the *First things First – get tested* campaign which was a collaborative effort between various organisations and implemented on campus by the Foundation for Professional Development. Both The Gentlemen’s Club and the *First things First – get tested* campaign aim to help South African students fulfil their destinies by encouraging them to be responsible, get tested for HIV and empower themselves by knowing their HIV status and committing to behaviour that will protect them and their peer group.^3^ To join the club, a student has to take a confidential HIV test so that he knows his HIV status before he fills in a membership form (which does not reveal his HIV status). The idea of a gentlemen’s club is borrowed from Steven G Peters, who had founded The Gentlemen’s Club in the USA and who had appeared on the Oprah Winfrey television show. According to him the aim of The Gentlemen’s Club is to transform young males into gentlemen so that they model a gentlemen’s behaviour in their communities and amongst their peers.^13,14^

The Gentlemen’s Club serves as a platform for male students to discuss sexuality and reproductive health issues such as HIV and AIDS. Motivational speakers are regularly invited to the club to address members on issues that would help shape them into responsible men. Club members support and motivate one another as they confront life’s challenges. As men are usually more influential and also usually act as decision makers on sexual matters in their relationships with women, their acceptance of HCT will influence their partners and friends, thereby increasing HCT uptake on campus. The club was initiated in May 2010 and was officially launched on 2 October 2010. 

HCT uptake for both males and females has shown an increase since the club has been launched. There has been an increase in the membership as well as in the number of students attending club meetings. A Ladies’ Club was launched late in 2011 to emulate The Gentlemen’s Club. Research to evaluate the impact of The Gentlemen’s Club on HCT uptake and other health problems affecting students, such as drug and alcohol abuse, condom use, STI and violence, will be performed by postgraduate students in health and social sciences. Because of its potential to increase and sustain HCT uptake, further support will be provided to the club to make it more attractive to other male students on campus.

## Discussion

The Gentlemen’s Club was launched at this university to increase HCT uptake. Innovative ways to communicate to students and mobilise them to have regular HIV test are always necessary as students get used to existing strategies. The two concepts of ‘gentleman’ and ‘club’ appeal to male university students as most of them are in the process of developing an identity and also have a need to belong. A gentleman is a person who observes certain rules of etiquette related to conduct and dress, and commands respect in society.^15^ A gentleman in the context of this health intervention is a male student who is committed to responsible sexual behaviour and is a role model to other students. The concept of a club, on the other hand, satisfies the need to belong for many male students on university campuses. The idea of The Gentlemen’s Club appeals to both male and female students, hence the launch of The Ladies’ Club. The rules and regulations of the club as well as regular speeches by motivational speakers make The Gentlemen’s Club a vehicle to address health and social issues facing university students. 
